# SIFT-GVF-based lung edge correction method for correcting the lung region in CT images

**DOI:** 10.1371/journal.pone.0282107

**Published:** 2023-02-28

**Authors:** Xin Li, Bin Feng, Sai Qiao, Haiyan Wei, Changli Feng

**Affiliations:** 1 College of Information Science and Technology, Taishan University, Taian, P. R. China; 2 College of Teacher and Education, Taishan University, Taian, P. R. China; Majmaah University College of Applied Medical Sciences, SAUDI ARABIA

## Abstract

Juxtapleural nodules were excluded from the segmented lung region in the Hounsfield unit threshold-based segmentation method. To re-include those regions in the lung region, a new approach was presented using scale-invariant feature transform and gradient vector flow models in this study. First, the scale-invariant feature transform method was utilized to detect all scale-invariant points in the binary lung region. The boundary points in the neighborhood of a scale-invariant point were collected to form the supportive boundary lines. Then, we utilized a Fourier descriptor to obtain a character representation of each supportive boundary line. Spectrum energy recognizes supportive boundaries that must be corrected. Third, the gradient vector flow-snake method was presented to correct the recognized supportive borders with a smooth profile curve, giving an ideal correction edge in those regions. Finally, the performance of the proposed method was evaluated through experiments on multiple authentic computed tomography images. The perfect results and robustness proved that the proposed method could correct the juxtapleural region precisely.

## I. Introduction

In the last three decades, lung cancer has become the deadliest cancer worldwide. Computed tomographic (CT) slices are considered the gold standard for lung disease diagnosis among various imaging modalities. Using CT slices to locate nodules is natural to detect lung cancer nodules earlier. A computer-aided diagnosis (CAD) system was developed to use a computer algorithm to detect lung nodules. The CAD system is considered a benchmark in lung cancer screening programs. It has helped achieve a 20% reduction in mortality [1].

In a CAD system, lung region segmentation is often used as the primary step in CT image processing because it reduces the analysis area and the computational resource consumption. Many algorithms [2–7] have been proposed for segmenting the lung region from CT images. The theoretical grounding of these methods is different Hounsfield unit (HU) value ranges for various organs [4]. However, the HU values of juxtapleural nodules are similar to those of their surrounding organs. And, it is different from those of the central lung region. Therefore, these juxtapleural nodules were omitted by the HU value threshold algorithms, which is a non-ideal result for the following steps. As a result, juxtapleural nodule regions are excluded from the extracted lung regions. Statistically, 5%~17% of pulmonary nodules are lost during the segmentation stage of the lung region [8].

According to the radiologist, juxtapleural nodules are more likely to be positive lung cancer nodules than other lung nodules. A lack of juxtapleural nodules significantly affected the accuracy and performance of the proposed method. To solve this problem, this study aims to design an algorithm to re-include juxtapleural nodule regions.

Many authors [9–12] have proposed their algorithms to address the omission of juxtapleural nodules in the lung region. Keshani et al. [13] and Sun et al. [14] used morphology templates to correct the lung boundary directly, omitting the finding step. However, selecting an appropriate model in the actual programming is challenging. Besides, many scholars have proposed algorithms to identify the region of Juxtapleural nodules based on different properties of edge curves. Yim et al. [15] supposed that juxtapleural nodules lie in the concave region of the lung boundary, and the single hollow area is an abrupt change in the boundary curvature. However, the result of the method may be worse when noise points exist along the border. Pu et al. [16] presented an adaptive border marching algorithm to include juxtapleural nodules while reliably minimizing the over-segmentation of adjacent regions. In this method, the detected points are connected by straight-line sections. Wei et al. [10] separated the entire lung into left and right lobes in the first step and calculated the chain code and lung contour to represent its boundary. Then, the concavity and convexity of the border are distinguished according to the chain code curve. Finally, the sunken edges are corrected using a filling step. Dai et al. [17] used the Graham method to obtain all convex points in the boundary when scanning along the contour line. Then, the convex point is used to calculate a cross-product that can help determine whether a region is concave. Finally, the detected hollow area was corrected using the concave hull method. Singadkar et al. [18] located concave and convex regions using dominant points and connected straight lines to restore the defective boundary line. Finally, manual participation and active contour model-based algorithms were also developed to correct lung regions. Messay et al. [19] proposed a fully automated (FA) and semi-automated (SA) system to segment lung nodules from CT slices precisely. In this method, a single user-supplied cue point or eight user-supplied control points must be offered by the user to obtain a more precise result. Although the precision of segmentation is high, it increases the user burden. Feng et al. [20] divided lung region into several regional blocks using a mesh grid. Then the fractal dimension values were calculated for each block. A block is recognized as a defective block using an adaptive threshold. The geometric active contour model is employed for a defective block to include the block in the lung region. A K-means cluster method [21] was introduced to acquire separated suspicious boundary lines.

In recent years, novel models and neural network-based methods have shown great potential in lung region segmentation. Kumar et al. [1] proposed an automated lung parenchyma segmentation method. The bidirectional code and the support vector machine (SVM) were used to improve the segmentation and reduce the false inclusion of regions correspondingly. Liu et al. [22] combined the wavelet transformation and a series of morphological operations to segment the lung region from a CT slice. The fast corner detection technique was then used to correct and smooth the extracted lung contours. Chung et al. [23] segmented the lung region with the Chan-Vese (CV) model and restored the lung region according to the previous frame images or the neighboring upper frame images. Seelan et al. [24] used the fuzzy threshold method to perform lung image binarization and lung region corrections. Jamshid et al. [25] improved the region-growing algorithm to achieve accurate segmentation of lung tumors while reducing the computational time of the algorithm and increasing the accuracy. Gan et al. [26] implemented a hybrid convolutional neural network (CNN) for automatic lung tumor delineation on CT images. Experiments on 260 cases demonstrate that the hybrid neural network can achieve good tumor segmentation performance on CT images. Jalali et al. [27] applied the improved U-Net model to lung segmentation. This deep model performed automatic segmentation of lung CT images by deep neural networks. Sahu et al. [28] used a 3D CNN to obtain a coarse segmentation of the right and left lungs. Another 3D structure correction CNN was then used to perform shape correction. Finally, the corrected regions were refined by a parallel flood-fill operation.

This study presents a novel boundary correction method that can include omitted juxtapleural nodule regions in the lungs. First, all scale-invariant feature transform (SIFT) points in a binary lung segmentation image were calculated. Then, the suspicious boundary lines are divided into several separate sublines when finishing the supportive boundary computation and connectivity analysis. Subsequently, the Fourier descriptor is used to characterize the boundary status of a suspicious subline, and a correction-needed boundary line is recognized by a spectrum energy threshold. Finally, the gradient vector flow-snake (GVF-Snake) model was adapted to correct the above boundary with a smooth contour line.

The main advantages of the presented method can be expressed as follows:

1. The spectrum energy of the Fourier descriptor performs better in recognizing non-ideal boundaries from all supportive edges. This term is a global property on the whole supportive curve, which is robust to noise and boundary curvature changes.
2. The GVF-Snake model introduces internal force energy, which acts as a smoothing constraint and ensures the smoothness of the final correction result. Further, the smooth results are more consistent with the actual state of the lung border than the linear connection methods.

The remainder of this manuscript is organized as follows. The details of the proposed method, including the recognition and correction steps, are discussed in Section 2. In Section 3, the numerical experiments on authentic CT images are presented. Finally, the conclusions of this study are presented in Section 4.

## II. Proposed method

### A. Main frame

**Fig 1:**
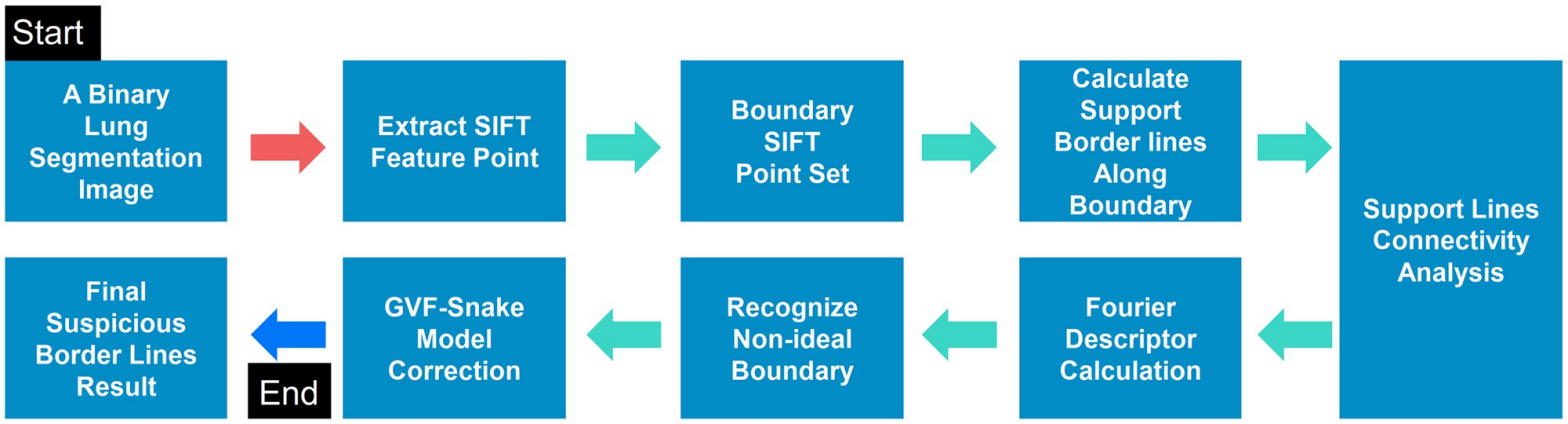
The main workflow of the proposed algorithm.

As shown in Fig 1, the proposed boundary correction method mainly consists of four main steps: SIFT point extraction, supportive edge calculation, Fourier descriptor recognition, and GVF-snake-based edge correction. Each part contains different sub-steps, which are explained as follows.

### B. Processing object

**Fig 2:**
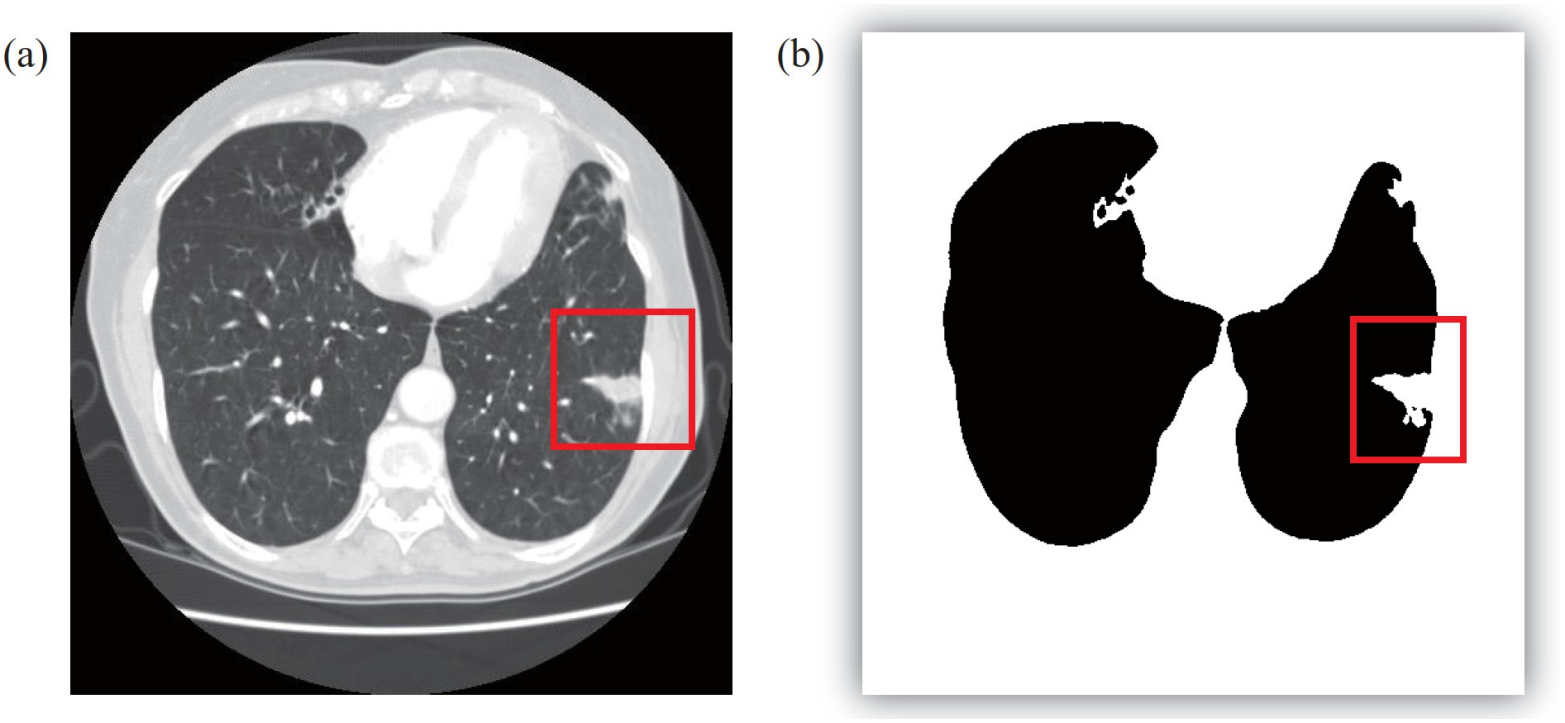
An example of the missed nodal area. (a) An original CT and (b) extracted lung region using a threshold method.

Compared with other auto-lung segmentation methods, this study focuses on correcting non-ideal boundaries. Other pre-processing steps are required in the first step of the proposed algorithm. Conversely, the process object of the proposed method, shown in Fig 2b, is the binary segmentation result obtained using the threshold method. Based on the rectangle box in Fig 2a, some juxtapleural nodules are excluded from the lung region in the CT segmentation result.

### C. SIFT point detection

For the lung binary image, the next step is to locate landmark points that can indicate the position of the juxtapleural nodule region. After analyzing the shape of the lung, most boundary lines are smooth, and the juxtapleural nodule is the intruder of the smooth status. Moreover, there is a vast difference in their curvatures. Motivated by this observation, the juxtapleural nodule region can be modeled as a rapid change in the curvature of the boundary lines.

This study introduced the SIFT method [29] to identify these boundary curvature changes. There are two critical reasons for the selection of this method. The first is that the SIFT feature can extract key points of objects and is invariant to uniform scaling, orientation, and illumination changes. Additionally, the SIFT feature has less power in detecting smooth lines, while it can extract corner points and endpoints well, which are the signals of concave regions.

The first step of the SIFT method is calculating the scale space of a lung image, which can be expressed as

L(x,y,σ)=G(x,y,σ)*I(x,y), (1)

Where *G*(*x*, *y*, *σ*) are the Gaussian functions with the variable parameter $\sigma \left(G\left(x,y,\sigma \right)=\frac{1}{2\pi \sigma^{2}}e^{-\frac{x^{2}+y^{2}}{2\sigma }}\right)$. The symbol * denotes the convolution operation at different scales. After this step, the difference operation was performed on successive Gaussian-blurred images.

Let *k* be the scale factor of two adjacent Gaussian scale-spaces. The difference of Gaussians (DoG) was calculated in this step. Using different scales, the key points are the minima and maxima of the DoG, which can be formulated as

$$\begin{array}{r} D\left(x,y,\sigma \right)=\left[G\left(x,y,k\sigma \right)-G\left(x,y,\sigma \right)\right]*I\left(x,y\right) \end{array}$$ (2)

$$=L\left(x,y,k\sigma \right)-L\left(x,y,\sigma \right),$$ (3)

where *L*(*x*, *y*, *kσ*) is the convolution of the original image *I*(*x*, *y*) with the Gaussian blur *G*(*x*, *y*, *kσ*) at scale *kσ*.

Unstable noisy vital points were observed after the calculation mentioned above. To deal with these points, a detailed fitting step was performed to filter out extreme noises. In the next level, each critical point was assigned one or more orientations based on the local image gradient directions when all noise is removed, which can be obtained through the following two terms:

$$m_{1}\left(x,y\right)={\left(L\left(x+1,y\right)-L\left(x-1,y\right)\right)}^{2},$$ (4)

$$m_{2}\left(x,y\right)={\left(L\left(x,y+1\right)-L\left(x,y-1\right)\right)}^{2},$$ (5)

$$m\left(x,y\right)=\sqrt{m_{1}\left(x,y\right)+m_{2}\left(x,y\right)},$$ (6)

$$d_{1}\left(x,y\right)=L\left(x,y+1\right)-L\left(x,y-1\right),$$ (7)

$$d_{2}\left(x,y\right)=L\left(x+1,y\right)-L\left(x-1,y\right),$$ (8)

$$\theta \left(x,y\right)=arctan\left(d_{1}\left(x,y\right),d_{2}\left(x,y\right)\right).$$ (9)

After completing the gradient calculation of key points, a histogram was built to count the gradients and directions of pixels in the neighborhood. The gradient histogram divides the directional range from zero to 360 degrees into 36 columns (bins), where each pin indicates 10 degrees.

**Fig 3:**
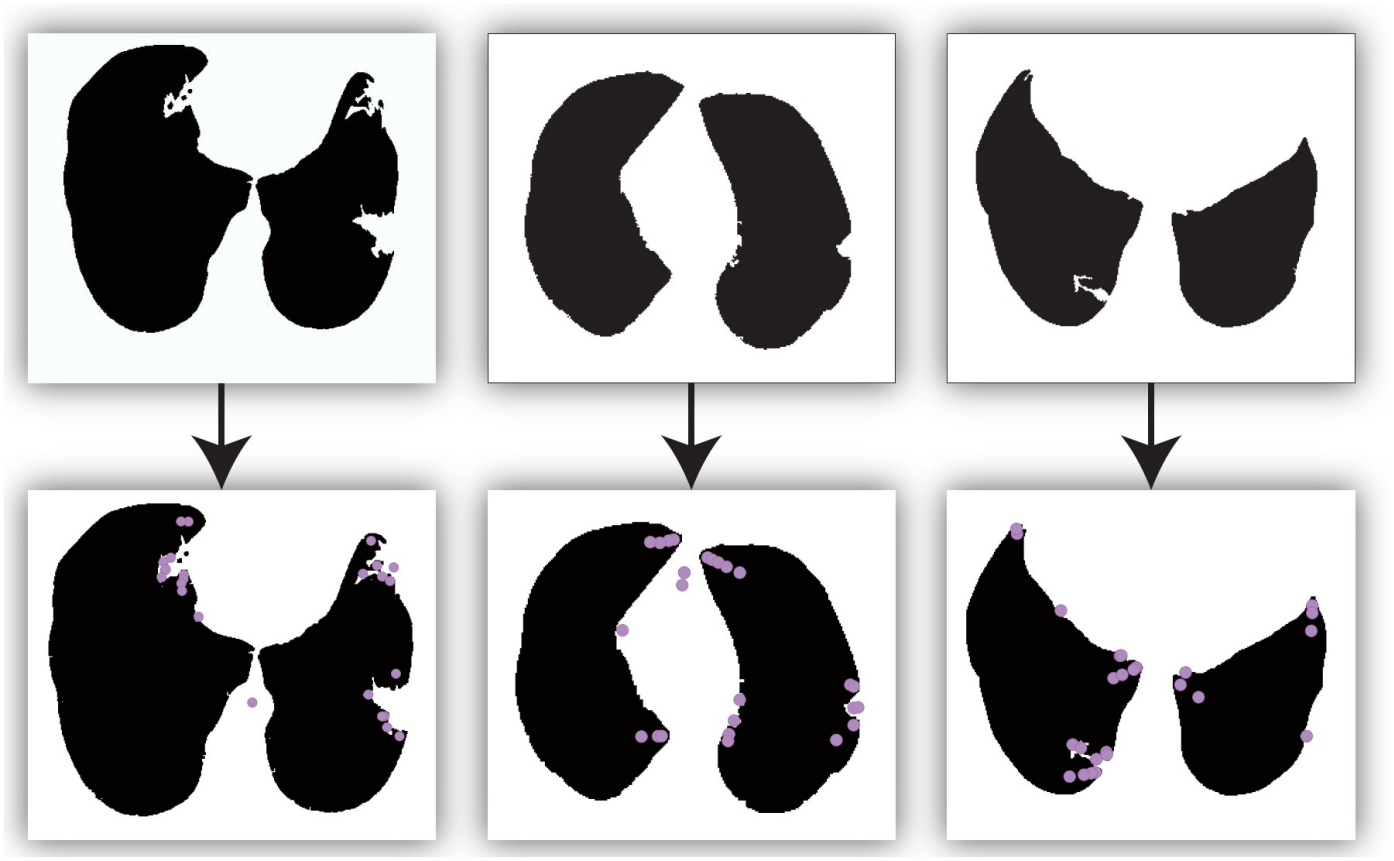
The detected SIFT points of the three images.

Finally, after the local histogram calculation and normalization, we obtained the final key point descriptor that can be used to detect the signal point along the lung boundary. As a result, all identified SIFT points are shown in Fig 3. Note that the result fits our assumption well, verifying the efficiency of the proposed method.

### D. Supportive boundary

Analyzing all SIFT points in Fig 3, most points are in the boundary line, but a few lie in the inner or outer region of the lung. Based on the assumption mentioned previously, we should focus on the curvature of the boundary, indicating that only the edge SIFT points are valuable data. Thus, dealing with these non-edge SIFT points is challenging.

We analyzed the positional relationship between the non-edge SIFT point and the suspicious region. Although the non-edge SIFT point is not in the boundary line, it is close to the edge whose curvature significantly changes. That is, these non-edge points are not useless, and they can provide some signals to identify suspicious regions. Therefore, we replaced the non-edge SIFT points with their closest boundary points. The following formula expresses this transformation:

$$f:P^{\prime }\mapsto P,P^{\prime }\in S,P^{\prime }\notin E,P\in E,$$ (10)

where *S* is the set of all SIFT points, *E* is the set of all boundary points, *P*′ is a non-edge point, and *P* is the closest point of *P*′ in set *E*.

All SIFT points were transformed into a new set S_e_, S_e_ = f pjf:p^0^∇ pg. This new set can determine the position of boundaries within the neighborhood of the feature point, which is called the supportive boundary line of suspicious areas. It is defined as

$$s\left(x,y\right)=\left\{\begin{array}{ll} 0, & if\sqrt{{\left(x_{i}-p_{x}\right)}^{2}+{\left(y_{i}-p_{y}\right)}^{2}}\leq R\\ 1, & if\sqrt{{\left(x_{i}-p_{x}\right)}^{2}+{\left(y_{i}-p_{y}\right)}^{2}}>R \end{array}\right.,$$ (11)

for *i* = 1, 2, 3, …, *n*, where *s*(*x*, *y*) is a point of a binary image in which there are only several supportive lines and (p_x_;p_y_) denotes a point of set *Se* and (x_i_;y_i_) is a point of the binary image.

**Fig 4:**
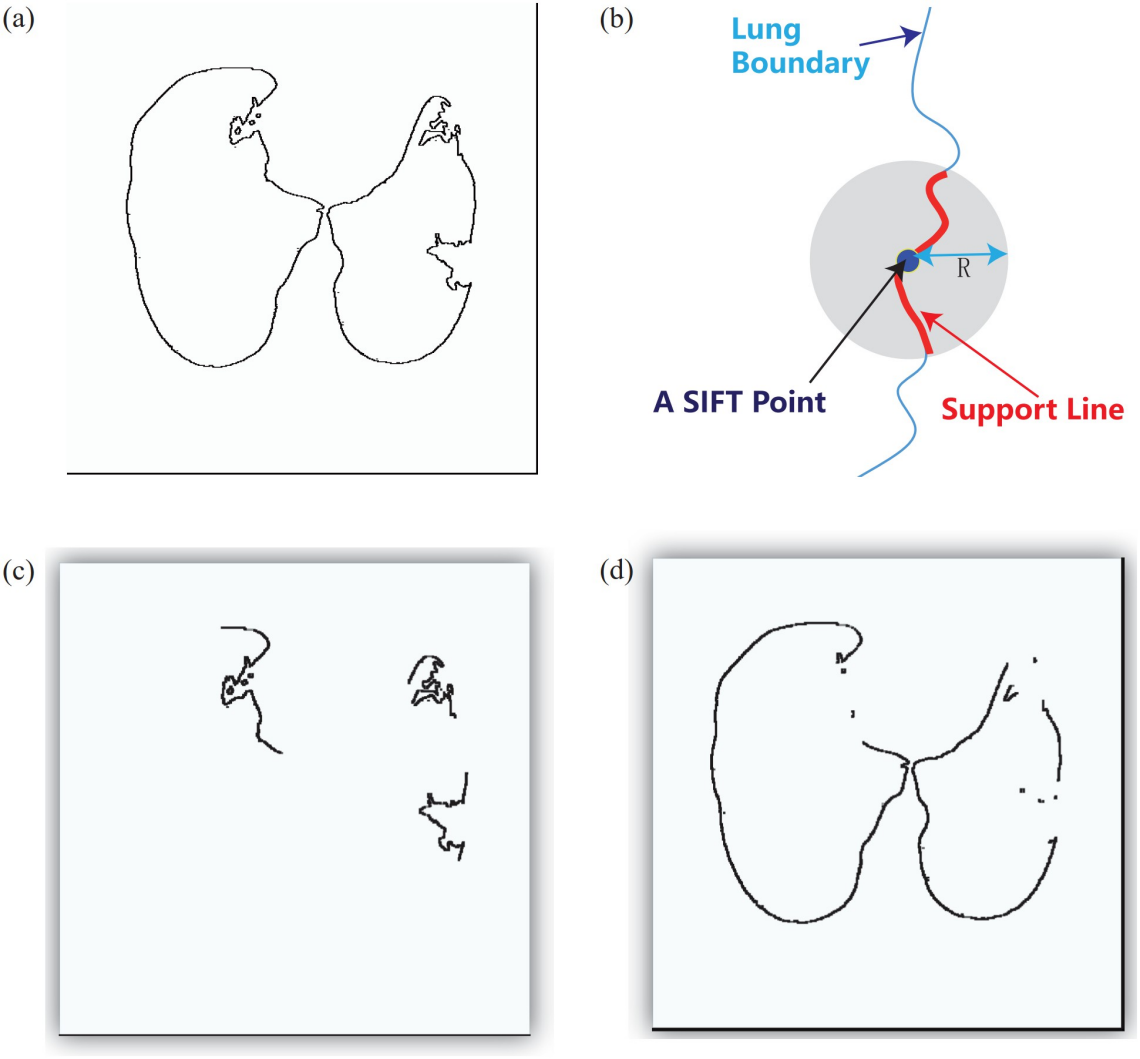
Supportive boundary lines and related results. (a) Whole lung boundary; (b) algorithm chart of the supportive line; (c) detected supportive lines; and (d) rest of the boundary.

Fig 4a shows all the boundary lines of the lung region; Fig 4b describes the principle of the supportive boundary algorithm. Fig 4c shows that a binary image containing only supportive lines was obtained by operating Eq (11) at every point in the image. Based on this figure, the supportive line consists of all points in the neighborhood of a SIFT edge point. In addition, all suspicious region lines are left, and all useless regular boundary lines are discarded by analyzing the lines in Fig 4d. In short, the supportive boundary extraction method defined in Eq (11) exhibits excellent performance in detecting suspicious regions.

### E. Recognition

The number of the obtained supportive lines is greater than two in the above results. However, in most cases, the number of actual juxtapleural regions is only one or two per slice. That is, some lines do not belong to the juxtapleural area. They may be regular lung edges whose curvature is larger than smooth edges but smaller than an actual juxtapleural boundary. Nevertheless, compared to actual suspicious juxtapleural borders, they should be ruled out from the regions that must be corrected. Therefore, developing a new model to recognize false-supportive border lines from all detected lines is necessary.

**Fig 5:**
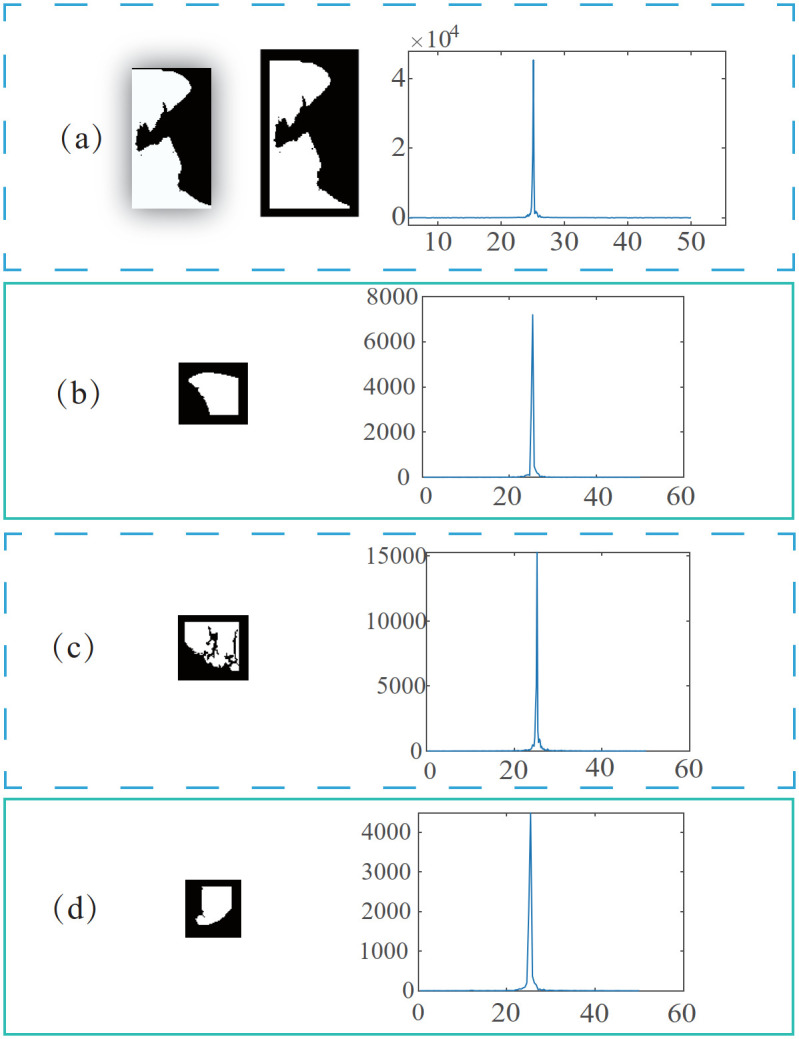
MERs and their Fourier descriptor figure. (a)-(d) show some local areas detected by the algorithm that need boundary repair and their Fourier descriptor figure.

We created a new method based on the Fourier descriptor to recognize these false supportive borders. As the primary step, the minimum enclosing rectangle (MER) of a given supportive line is calculated to include the inner and outer lung points. The rectangle is also called the original rectangle (OR). Subsequently, we added the outskirt background around OR by extending the rectangle size. In the extended region(ER), the OR is at its center, and the expanded background points are at its outskirts. This step mainly aims to generate an enclosing edge for the supportive line. An example of this step is shown in the first line of Fig 5, in which Fig 5a and 5b show the OR and ER, respectively.

Let {(*x*(*n*), *y*(*n*))|*n* = 1,2,3, …, *N*}denote the coordinate set of all the points on the closed boundary of the ER. Its complex expression can be written as

$$z\left(n\right)=x\left(n\right)+jy\left(n\right),n=1,2,3,\cdots ,N.$$ (12)

The original 2D border is closed, and its one-dimensional form has periodicity with a period of *N*. Under this observation, the discrete Fourier transform can be defined as

$$Z\left(k\right)=\sum_{n=0}^{N-1}z\left(n\right)exp\left(-\frac{j2\pi kn}{N}\right).$$ (13)

The result obtained from Eq 13 is sensitive to displacement, rotation, scale, and starting point. To solve this problem, the coefficient *Z*(0) is set to 0 to make *Z*(*k*) robust to the displacement, rotation, and starting point. Then, every element of *Z*(*k*) is divided by *Z*(1). Its formula is expressed as follows:

$$Z^{\prime \left(i\right)}=\frac{Z\left(i\right)}{Z\left(1\right)},i=1,2,3,\cdots ,N-1.$$ (14)

The newly obtained series is called the Fourier descriptor of ER.

According to the properties of the Fourier descriptor, the lower frequency in *Z*(*k*) represents the overall shape of the object in the ER, and the higher frequency in *Z*(*k*) reflects the details of the object in the ER. It is easy to find whether there is a slightly significant difference when analyzing all extracted ER. Based on the above analysis, selecting a higher-frequency value for recognition is natural.

The figure of *Z*′(*n*) is plotted in Fig 5, where the block image on the left is the OR of every supportive line, and the figure of each row on the right shows its corresponding Fourier descriptor. Fig 5a shows a massive value between the 20th and 30th numbers. This position can be considered as a higher frequency. It represents a sudden change in curvature. Note that there are two more significant values in Fig 5a and 5c and two smaller ones in Fig 5b and 5d. We concluded that the value would be higher than 10000 when drastic curvature changes occur.

Additionally, the amount of 10000 has been verified in many experiments. Thus, we selected 10,000 as the threshold. That is, a block is considered a region in need of correction when its maximum Fourier descriptor value is greater than 10000.

### F. Correction

This subsection proposes a modified GVF-Snake model to correct the detected blocks. First, a rectangular line is set in the surroundings of the object in the ER. Then, an evolution equation lets the line move toward the inner line while simultaneously keeping the line continuous and smooth. When a line point arrives at the boundary of an object, the evolutionary curve stops moving. The area inside the final evolution curve is the corrected local block.

Based on the GVF-Snake model, we adapted the force balance equation as

$$F_{int}+F_{ext}^{g}+F_{ext}^{n}=0,$$ (15)

where *F*_*int*_ is the internal force, $F_{ext}^{g}$ is the GVF force, and $F_{ext}^{n}$ stands for a balloon force. Each term can be defined as

$$\left\{\begin{array}{ll} F_{int} & =\lambda_{1}E_{line}+\lambda_{2}E_{edge}\\ F_{ext}^{g} & =\kappa_{1}V\left(x,y\right)\\ F_{ext}^{n} & =-\kappa_{2}N\left(x,y\right) \end{array},\right.$$ (16)

where *κ*_0_, *κ*_1_, *κ*_2_ > 0 are the weighting parameters of the internal, GVF, and balloon forces, respectively. Further, *N*(*x*, *y*) denotes the outward unit normal vectors, and $F_{ext}^{n}<0$ deflates the evolution curve to the lung boundary because the original curve is outside the object.

Terms *E*_*line*_ and *E*_*edge*_ in the first equation (Eq 16) can be calculated using the following formula:

$$\left\{\begin{array}{ll} E_{line} & =I\left(x,y\right)*G_{\sigma }\\ E_{edge} & =-{\left|\nabla I\left(x,y\right)\right|}^{2} \end{array}\right..$$ (17)

**Fig 6:**
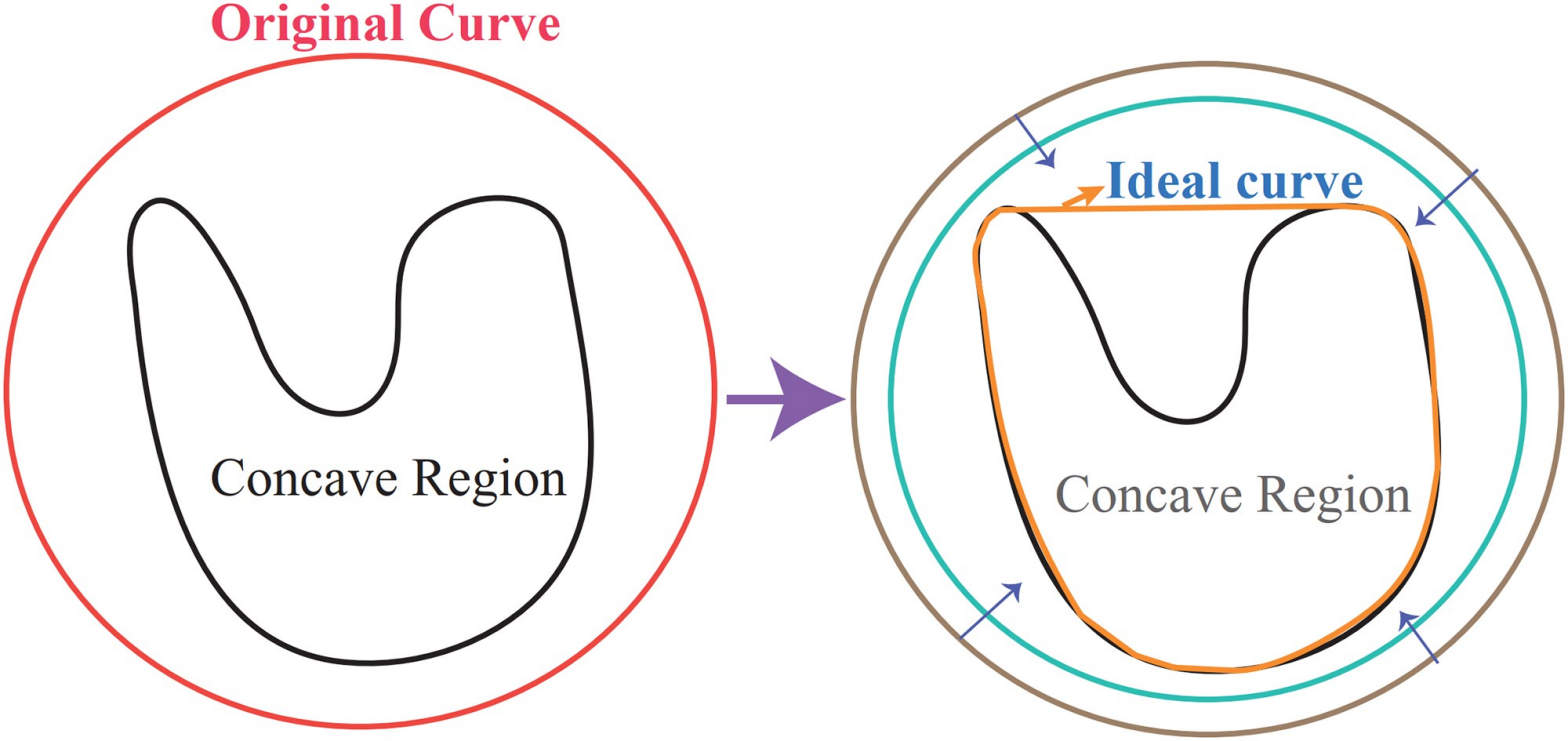
The principle of GVF correction.

Note that energy *F*_*int*_ does not contain a corner energy term. Although F_int_ can extract the evolution curve at the corner of an object, it is useless in the presented model. The reason is that corner points always appear in the suspicious region, but they are not in the interest of the proposed algorithm. To accommodate the absence of F_int_, we first set the original curve outside the object and deflated it to the object boundary, as shown in Fig 6.

Eq 15 can be re-written as

$$F_{int}+\kappa_{1}V\left(x,y\right)-\kappa_{2}N\left(x,y\right)=0.$$ (18)

Let the evolution curve be *X*(*s*) = [*x*(*s*), *y*(*s*)], *s* ∈ [0, 1]. *X* is a function of time, *t* and *s*. The partial derivative of *X* is then expressed as

$$X_{t}\left(s,t\right)=\lambda_{1}X\prime \prime \left(s,t\right)+\lambda_{2}X\prime \prime \prime \left(s,t\right))+\kappa_{1}V+\kappa_{2}N.$$ (19)

By using the matrix form, the solution of Eq 19 can be described as

$$\left\{\begin{array}{ll} x_{t+1}= & {\left(A+\gamma I\right)}^{-1}\left(\gamma x_{t}-\kappa_{1}V_{x}\left(x_{t},y_{t}\right)\right.\\ & \left.+\kappa_{2}N_{x}\left(x_{t},y_{t}\right)\right)\\ y_{t+1}= & {\left(A+\gamma I\right)}^{-1}\left(\gamma y_{t}-\kappa_{1}V_{y}\left(x_{t},y_{t}\right)\right.\\ & \left.+\kappa_{2}N_{y}\left(x_{t},y_{t}\right)\right) \end{array}.\right.$$ (20)

After the curve evolution based on Eq 20, we obtained a closed curve similar to the ideal trajectory in the right side subgraph of Fig 6. The inner part of the curve is considered the corrected region. However, the dealt object is the ER, only the area corresponding to the OR is our concern. Thus, the corresponding part of the OR, called the OR correction region (ORCR), should be extracted first. Additionally, in many experiments, some points that belonged to the lung region before correction were transformed into a member of the background. The main reason is that the used GVF-Snake model is not convex. It may stop at some local optima and not arrive at the global optimal, causing the above phenomenon to occur. To address this problem, the position of every lung region point is recorded before correction. Each is set to a lung region point again if they are transformed by mistake.

Finally, we replaced all MER regions with the ORCR in the binary lung regions. When all the “bad” blocks were fixed, the CT slice was rectified.

## III. Experiments

In this section, experiments are conducted on several real CT slices, and the experiments can be categorized into the following classes: (1) experiments on the lung image database consortium (LIDC) [29], (2) contrast experiments over other models, and (3) 3D reconstruction of correction results. The experiments were performed on a Dell Precision graphics workstation T5610, with two Intel Xeon E5-2630 CPUs, 32GB of internal storage, a 256GB SSD, and an Nvidia Quadro K4000 GPU. The parameter that is used in the manuscript is as follows: *λ*_1_ = 0.04, *λ*_2_ = 3, *κ*_1_ = 0.5, *κ*_2_ = 0.001, and *σ* = 1.2, *γ* 0 = 1.

The CT slices processed in the following experiment are the data of a patient chosen from the LIDC database. We selected 35 subjects containing juxta-pleural nodules. Each subject has more than 140 CT slices. These data were acquired by multiple detector computed tomography scanners. For example, the CT subject in Fig 2 contains a total of 196 CT slices acquired from the CT equipment of GE Medical Systems. The thickness of each slice is 2.5 mm. Other information is not available for privacy reasons. The second CT slice in Fig 3 shows another CT set, whose layer thickness of each CT slice is 2mm; the total number of all CT slices is 141. The clinically diagnosed condition is gating pulmonaire. Other private data is also deleted for the privacy policy. It is worth noting that the proposed algorithm is modeled to repair the pulmonary boundary, so this experiment will focus on correcting the boundary rather than showing the gating pulmonaire.

**Fig 7:**
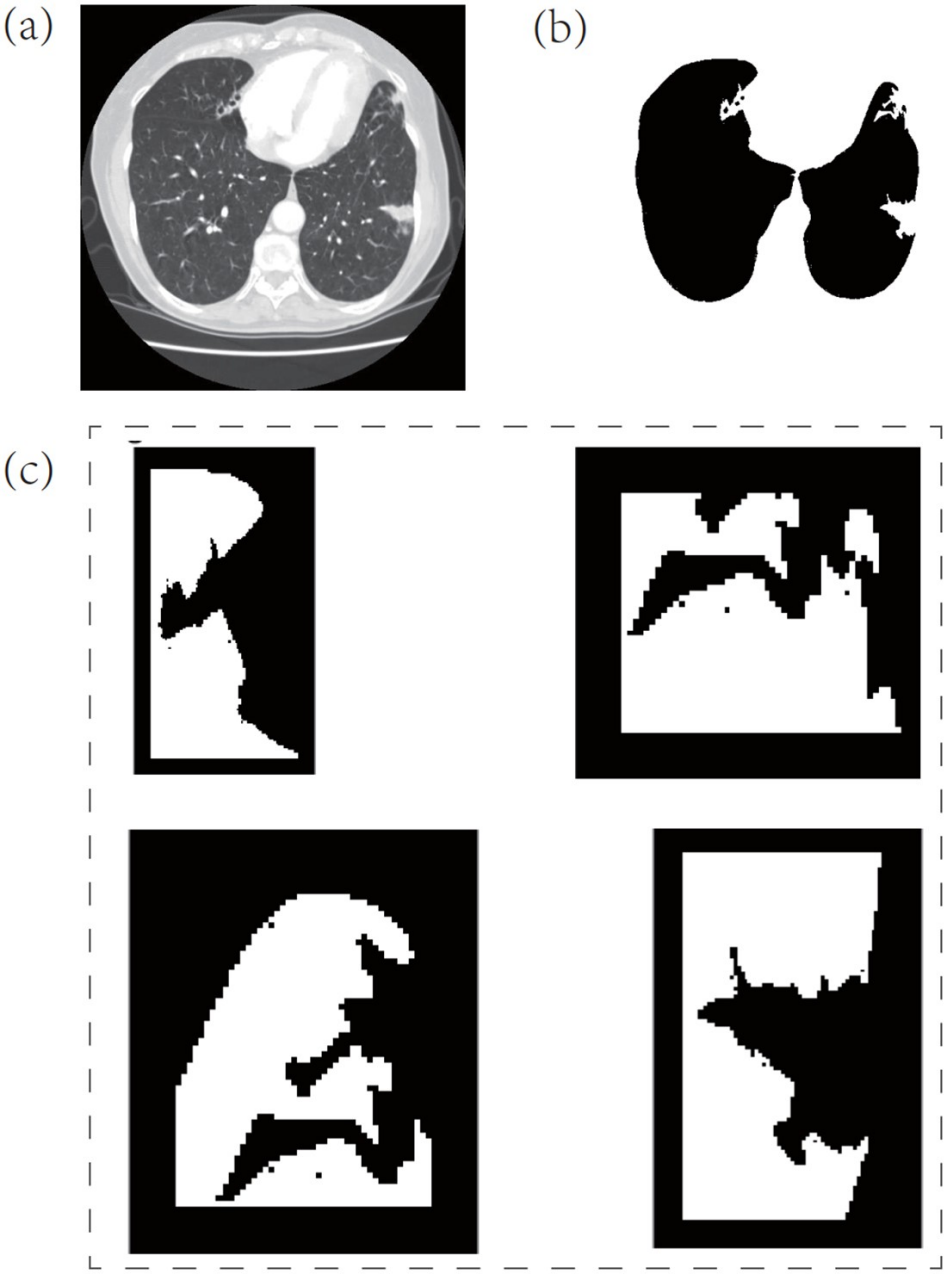
The detected blocks. (a) The original CT slice; (b) the initial lung region extracted by the algorithm; (c) the local blocks detected by the algorithm that needs boundary repair.

First, the CT slice in Fig 2 was tested by the proposed algorithm. Fig 7a shows the original CT image, and Fig 7b gives the extracted binary lung region by the threshold method, exhibiting that some omitted areas are caused by the disadvantage of the threshold method used. The four figures in Fig 7c are the detected MERs, from which it is known that all these regions are distilled using the proposed Fourier descriptor method. Any other suspicious MERs that required to be corrected were also excluded. All the above results prove that the Fourier descriptor and the selected threshold have a powerful ability to recognize “bad” boundary lines.

**Fig 8:**
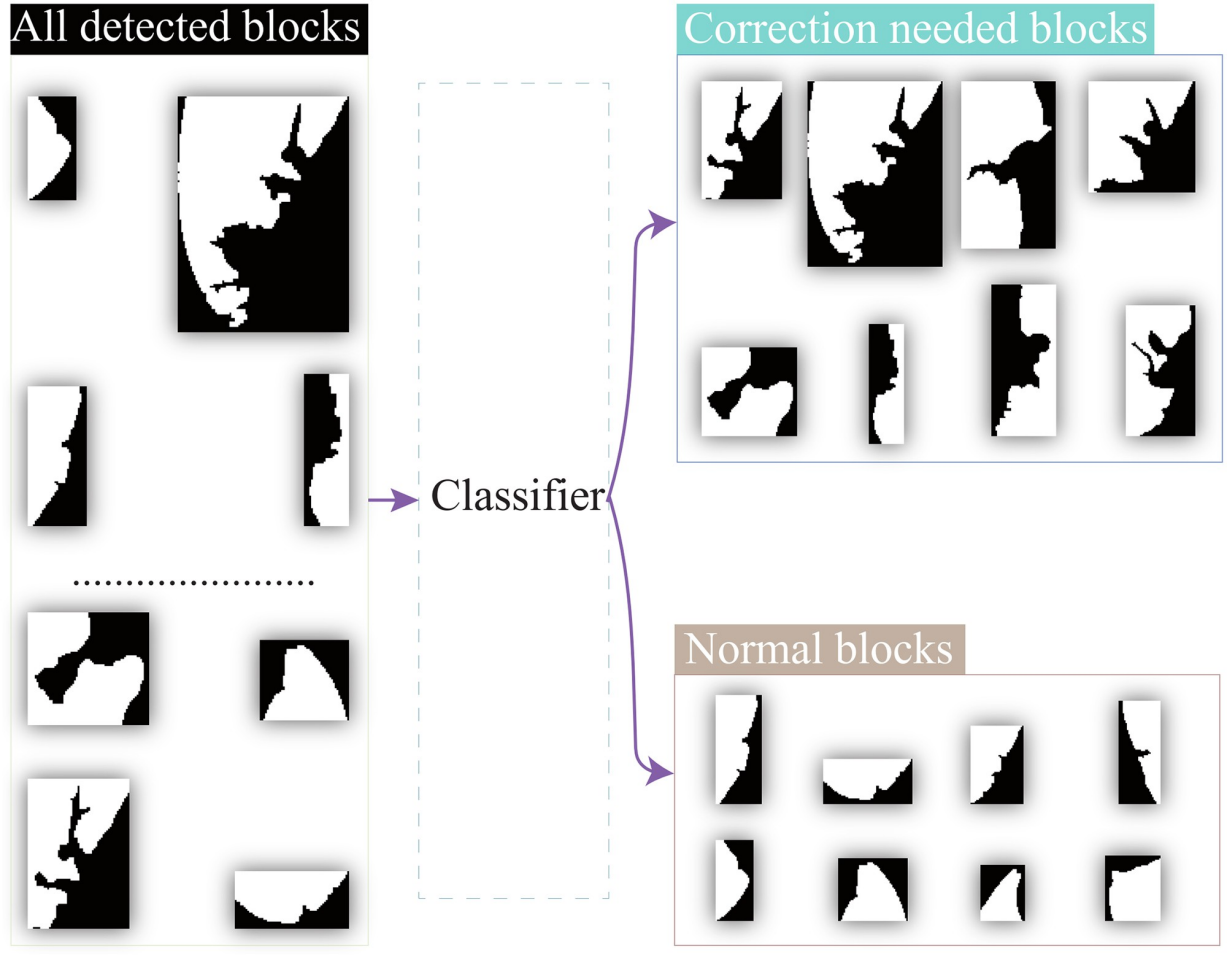
Classification of the two types of blocks.

Fig 8 shows the experimental results of the “bad” block identification method proposed in this paper. The blocks on the left are detected blocks containing normal and “bad” blocks. It is seen that the “bad” blocks have been identified from the normal blocks by the Fourier descriptor classifier. This classification result also agrees with the human manual recognition result. This figure also proved that the Fourier descriptor classifier has good block recognition ability.

**Fig 9:**
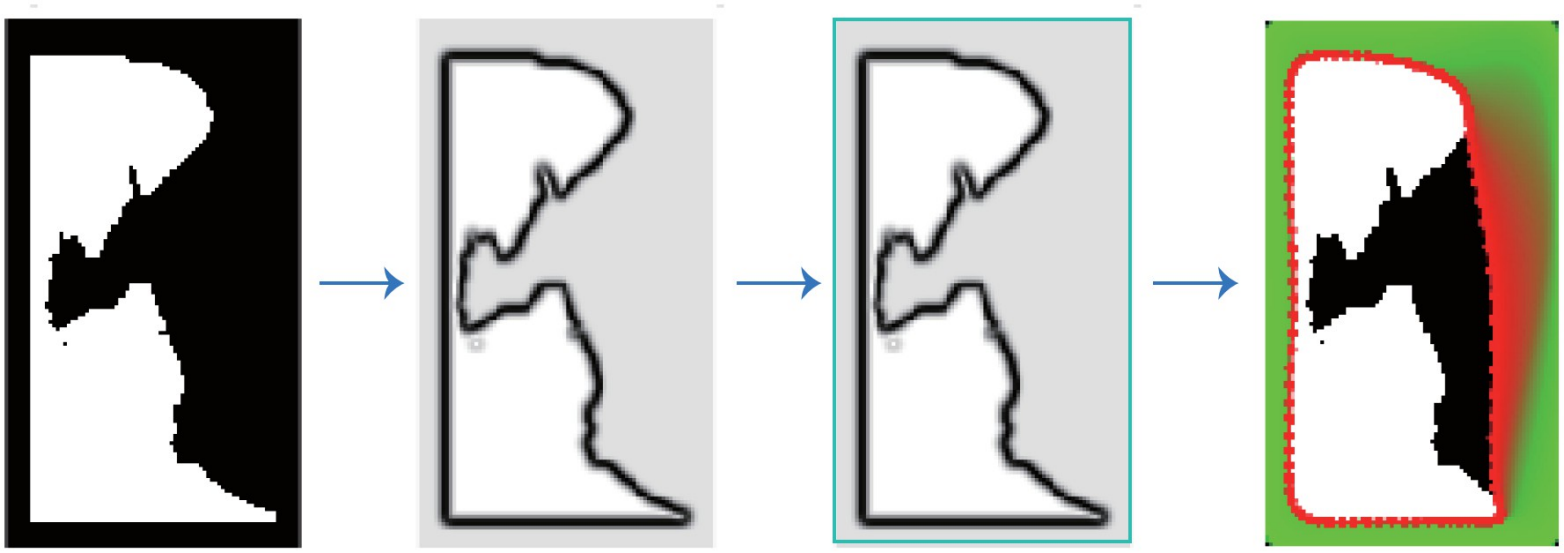
The correction on an MER.

**Fig 10:**
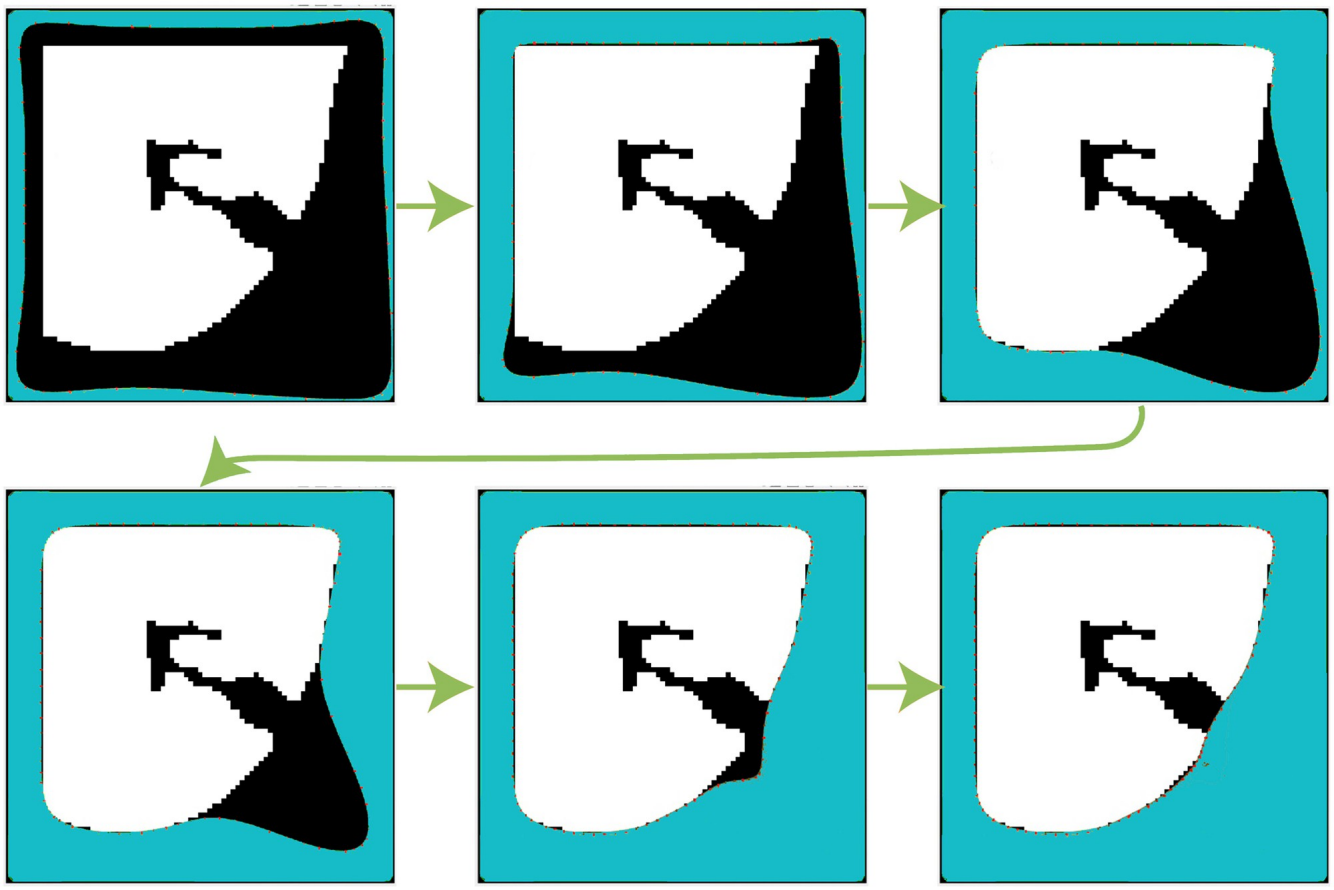
The overall boundary correction process for a local region.

Fig 9 shows the correction process results. The first sub-figure gives the initial contour used in this case, which is set as a wide black frame around the white object. The second one provides an image of external energy *F*_*int*_, and the third one displays the energy distribution of another external energy $F_{ext}^{g}$. The boundary of the third one denotes the initial rectangular outline of the correction model. The fourth one exhibits all the curve evolution processes in the ER correction, where the red points show the final corrected curve by the GVF-Snake model, and the green color denotes the position of the evolving curve points. Note that the evolution curve moves from the initial to the final position, stopping at an ideal location while keeping the curve smooth and elastic. Meanwhile, we showed the details of the curve evolution in Fig 10. In this figure, the white object is the local block detected by our recognition algorithm. The blue color denotes the region where the contour line has gone before the current iteration. The interior zone of the blue region in the last sub-figure is the corrected local block, in which the black area is the re-included part in this edge correction procession. From Fig 10, it is easy to find that the evolutional curve behaves like what we designed in the modeling phase. The repair process is more logical to human behavior; the repair results are smoother and more accurate, better reflecting the actual state of the lung edge.

**Fig 11:**
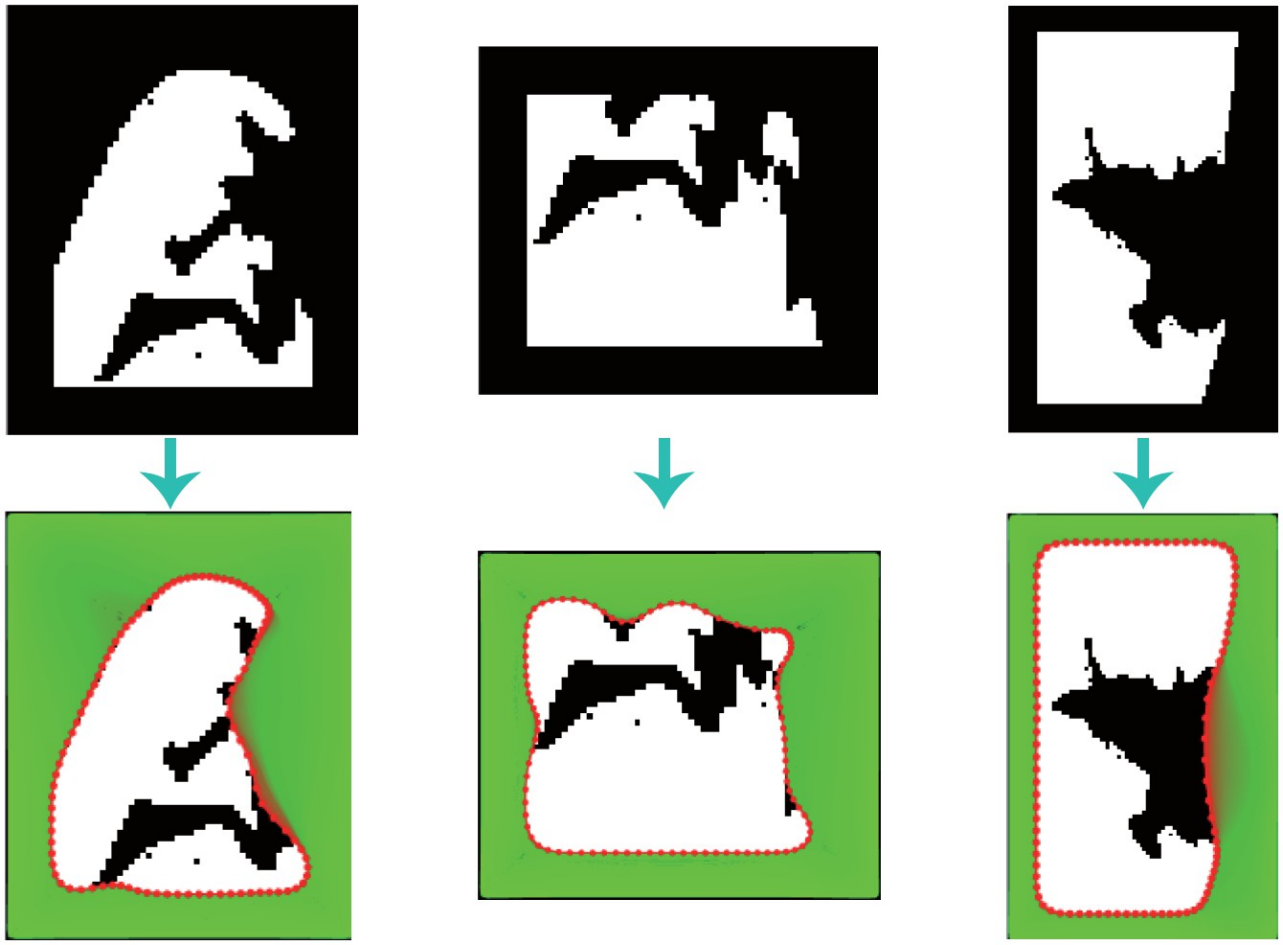
The correction effect of three local areas.

Fig 11 shows the corrected effect of another three ERs. These results in the second line demonstrate that the proposed GVF-Snake model is not sensitive to the shape of ER. Most “bad” boundary line sections are successfully corrected. The above results demonstrate that our model is robust, feasible, and reliable.

**Fig 12:**
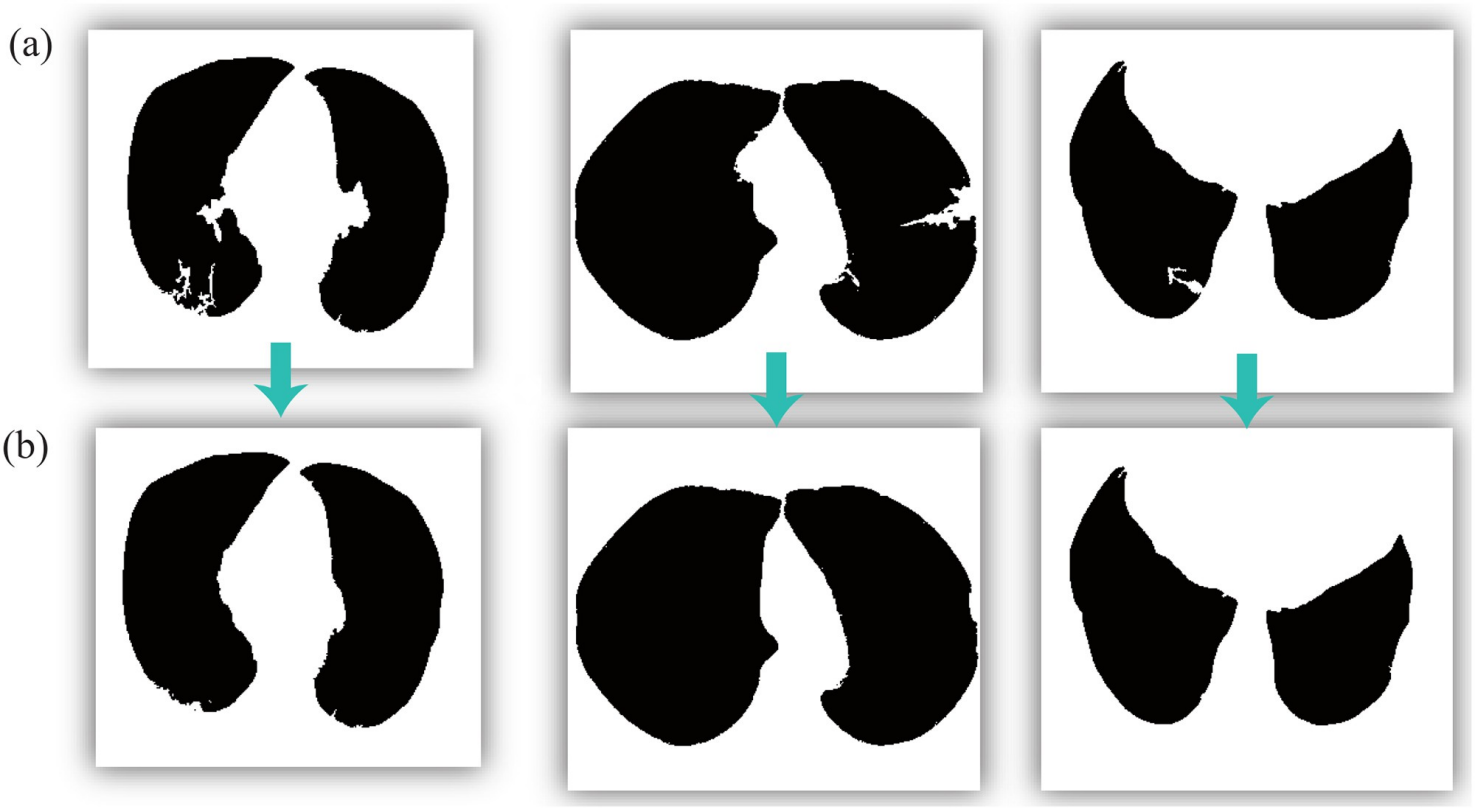
Binary image correction effect. (a) Initial lung regions and (b) corrected lung images.

The proposed model was evaluated using other CT images. The experimental results are shown in Fig 12. In this figure, all three images in Fig 12a have suspicious local regions along the original edge. In addition, in Fig 12b, these suspicious local regions are successfully corrected after using our proposed model. Finally, we obtained three ideal entire lung regions.

**Fig 13:**
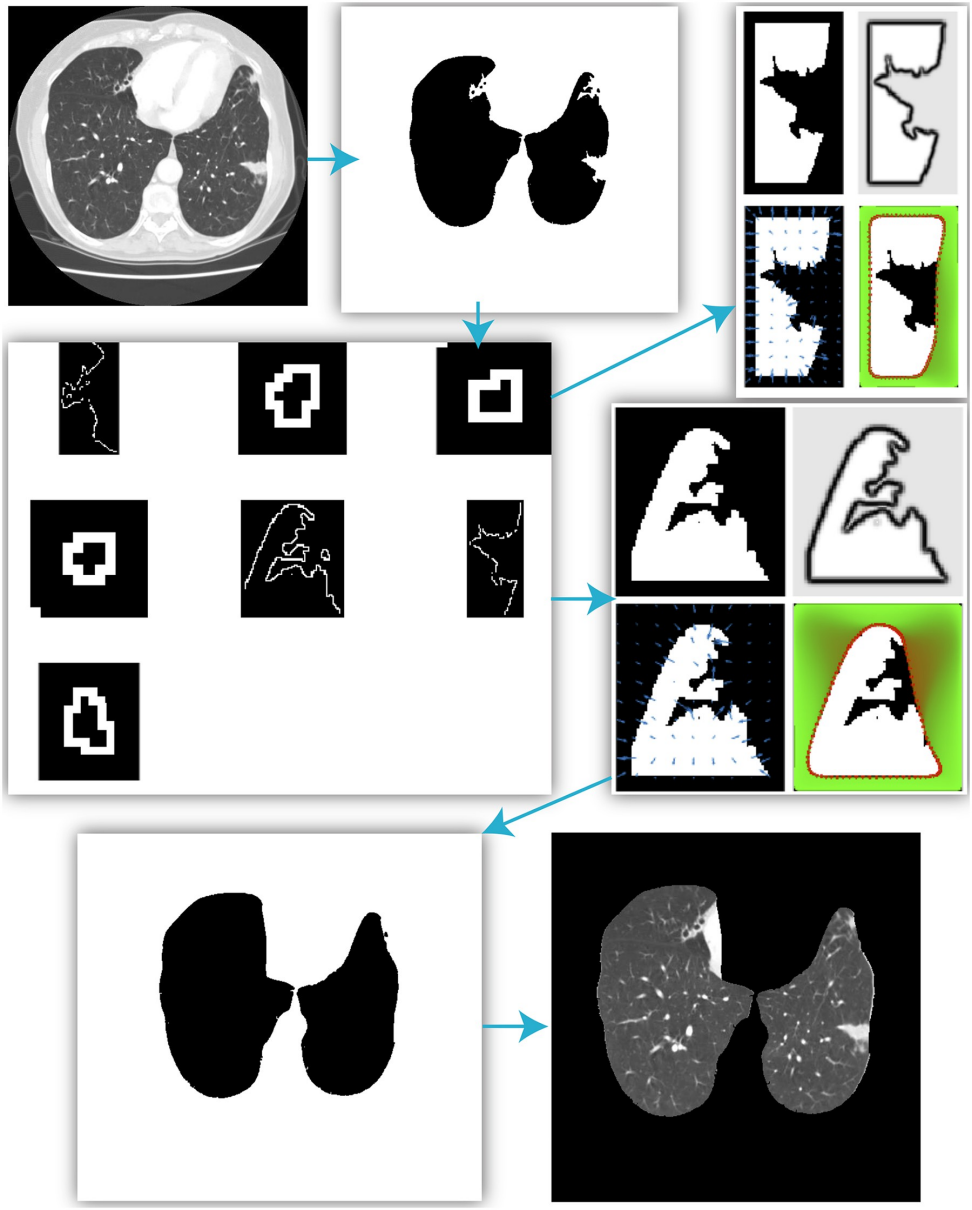
The segmentation results in the CT slice I.

**Fig 14:**
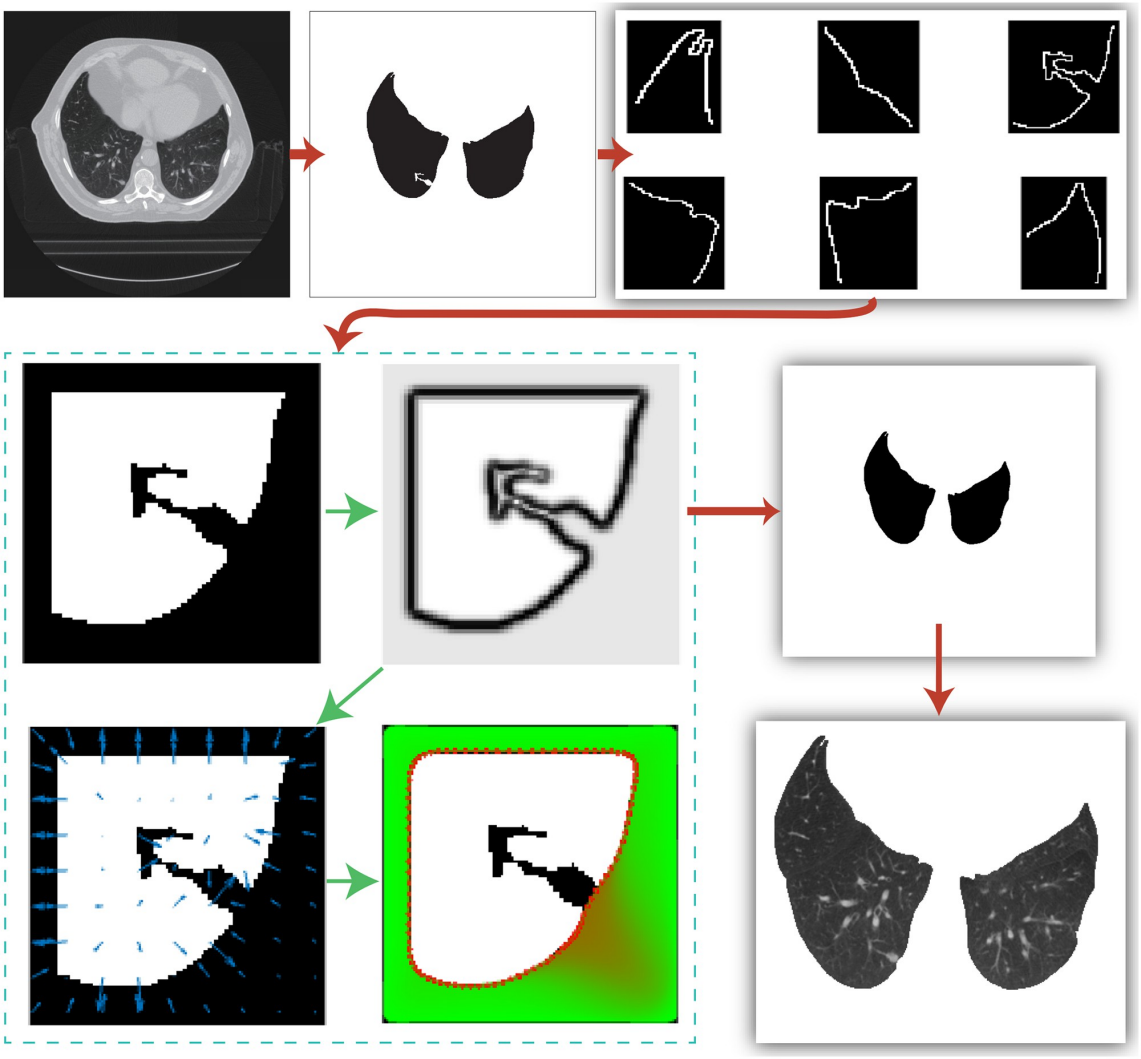
The segmentation results in the CT slice II.

To demonstrate the whole repair process of the proposed method in one figure, we summarized the essential results of two CT slices in Figs 13 and 14, respectively. The proposed algorithm successfully identified and corrected the “bad” local blocks and finally extracted the precise lung region from these results.

A severe problem was mentioned in the original manuscript [20] when conducting the experiment using the model described. The problem is that the correction result depends on the grid layout significantly. In some cases, the concave region in the right lung is divided into two blocks. Consequently, the correction result under this layout will be worse. The same operation was executed again to obtain a more suitable grid layout and correction results. However, to avoid the above ideal layout, the authors designed a second correction step for every case, making the model less efficient and nearly twice the calculation time. Because the grid is set based on a fixed strategy, the grid layout may still not work well in the second correction in some cases. In contrast, the model proposed in this study avoids this problem, and the correction step takes only half the time of the prior method.

**Fig 15:**
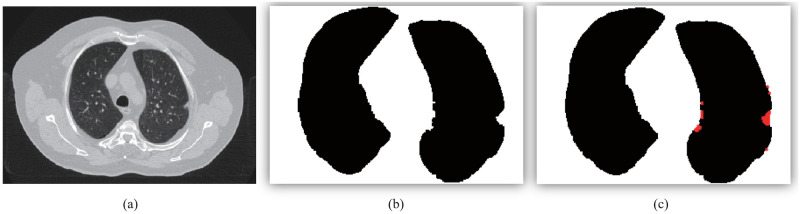
The correction effect comparison chart. (a) The original CT slice; (b) the extracted coarse lung binary region; (c) the corrected lung region.

Fig 15 provides the correspondence of the corrected region with its original CT slice. From Fig 15a–15c are the original CT slice, the segmented initial lung region and the corrected lung region, respectively. We rely on the serial number of the CT slice to correspond the corrected binary result to its original CT slice. If the corrected region contains suspicious nodules, the original CT slice where the nodules are located can be queried by the image number.

**Fig 16:**
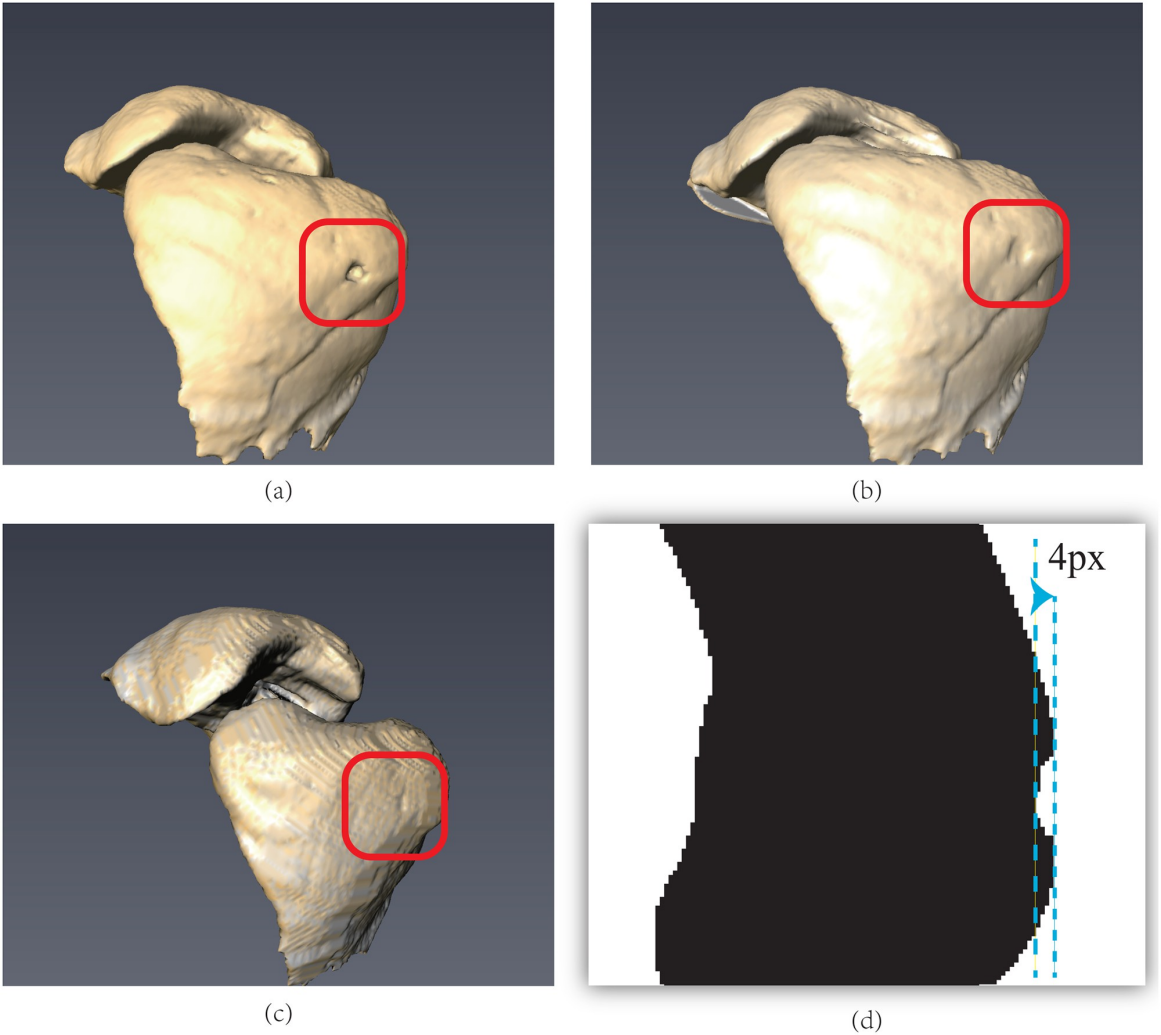
The comparison over 3D reconstruction models. (a) The model based on original CT slices; (b) the 3D model of this manuscript; (c) the reconstruction model of [18]; (d) the error of the algorithm in this paper.

To intuitively compare the corrected region, all 141 slices of a patient selected from the LIDC database were reconstructed to form a 3D model. The 3D result is shown in Fig 16, where there is an apparent concave hull in the left lung in Fig 16a. As shown in Fig 16b, the proposed algorithm successfully filled the concave hull. Although the filling effect in Fig 16c does not achieve the ideal conditions, and there remains a small hollow region in the reconstructed model, the maximum error from the corrected result to the ideal edge is only four pixels in depth, as shown in Fig 16d. This error is minor in other adjacent slices ranging from 1px to 2px. If these distances in the CT slice are transformed into the spacing distance, it equals 1 ~ 3.8 mm. These error values were quite small to impact the other algorithms compared with the standard size used in recognizing lung nodules. From this perspective, the correction results were acceptable.

In Fig 16c, the corrected 3D model based on the algorithm is also shown, from which it is easy to find two drawbacks. The first one is the unsatisfactory filling of concave areas, which is labeled with a red rectangle in the figure. The second one is the linear texture on the lung surface. Further, after rotating the position and angle of the camera, a similar linear texture remained on the 3D lung surface. Conversely, the results of the proposed model in this study maintain the same appearance in most regions.

This comparison of the 3D model demonstrates that the proposed model in this paper can correct the lung edge while maintaining the details of other lung edges. Therefore, the proposed SIFT-GVF-based model has a better correction ability.

Finally, experiments on the correction accuracy were also performed on CT slices to evaluate the effect of the proposed model. The experimental results were compared with other counterpart correction methods, including the Frac-Snake-based and morphology template-based models, to make the assessment result quantitative. In every type of model, the correction accuracy, under-correction accuracy, and over-correction accuracy were calculated based on the corrected results of every model.

The mean accuracy results are presented in Table 1, in which ACC denotes the percentage of the effective areas in the corrected results. R_u_ and R_o_ are the ratio of under-corrected pixels and over-corrected pixels, respectively. The following formula can calculate these two indicators:

$$ACC=\left|\frac{S_{manu}\cap S_{auto}}{S_{manu}}\right|\times 100\%$$ (21)

$$R_{u}=\left|\frac{S_{manu}-(S_{manu}\cap S_{auto)}}{S_{manu}}\right|\times 100\%$$ (22)

$$R_{o}=\left|\frac{S_{auto}-(S_{manu}\cap S_{auto)}}{S_{manu}}\right|\times 100\%$$ (23)

Where S_manu_ and S_auto_ represent the results repaired by the expert and the result corrected by the proposed model, respectively. From the data in the table, it is clear that our proposed algorithm obtains the highest accuracy. In addition, the algorithm proposed in this paper has the lowest *R*_*u*_ value, although it has a high *R*_*o*_ value. Therefore, on the whole, the algorithm proposed in this paper has the best performer among the algorithms in Table 1. And the proposed method can yield more precise correction results than the other four methods.

**Table 1:** Accuracy comparison between the proposed algorithm and existing algorithms.

*Indicator*	Method^1^	Method^2^	Method^3^	Method^4^	Method^5^
ACC	98.2%	97.8%	88.9%	87.4%	81.4%
R_u_	0.7%	0.4%	9.5%	11.7%	17.1%
R_o_	4.0%	4.3%	0%	0%	0%

^1^The method proposed in this study;

^2^the method proposed in [18];

^3^the rolling-ball method with a 5px radius;

^4^the rolling-ball method with a 10px radius;

^5^The rolling-ball method with a 15px radius.

In comparison, the deep network-based repair methods require a larger amount of data for training and consume more computational resources. These methods are more demanding on the hardware platforms. And the results of the linear connection-based methods are not smooth enough, which do not match the real state of the lung boundary, and the repair accuracy cannot be guaranteed. In contrast, the algorithm proposed in this paper first searches for suspicious regions along the lung boundary using SIFT information. It then identifies the regions to be repaired based on the threshold value. The algorithm process is very natural and consistent with human cognition. In addition, the GVF-Snake-based repair method can guarantee the smoothness of the border while repairing the lung border. This feature makes the repair result more consistent with the real state of the lung, thus obtaining a higher repair accuracy. Further, the average time to correct a binary lung region in the experiment was about 10 seconds. Taking the training time into account, the time consumption of the proposed method in this paper is still advantageous compared to the deep learning algorithm.

## IV. Conclusion

Juxtapleural nodules were often excluded from the segmented lung region of the HU threshold-based method. To re-include those regions from the segmented binary image, a new edge correction method was proposed based on the scale-invariant feature transform information, the supportive edge identification and the GVF-Snake model. First, the SIFT method was utilized to find all scale-invariant key points in the binary image. Second, the supportive edge was calculated according to the distance relationship between the scale-invariant key points and the edge points. The Fourier descriptor was then used to characterize the supportive boundary. The supportive boundary to be corrected was recognized using an adaptive threshold to the Fourier descriptor. Third, an initial curve was set around the identified local block, and the curve evolved according to the GVF-Snake model to gradually repair the lung edge. After the curve evolution has stopped, the suspicious edge has been successfully repaired. The proposed model exhibited good edge identification and edge correction capability through testing on multiple sets of clinical CT data.
